# Cell wall properties play an important role in the emergence of lateral root primordia from the parent root

**DOI:** 10.1093/jxb/eru056

**Published:** 2014-03-11

**Authors:** Peter S. Roycewicz, Jocelyn E. Malamy

**Affiliations:** Department of Molecular Genetics and Cell Biology, The University of Chicago, Gordon Center for Integrative Science W519, 929 East 57th Street, Chicago, IL 60637, USA

**Keywords:** Cell wall, developmental plasticity, extensin, lateral root, root system development, root system architecture, XEG113.

## Abstract

Plants adapt to their unique soil environments by altering the number and placement of lateral roots post-embryonic. Mutants were identified in *Arabidopsis thaliana* that exhibit increased lateral root formation. Eight mutants were characterized in detail and were found to have increased lateral root formation due to at least three distinct mechanisms. The causal mutation in one of these mutants was found in the *XEG113* gene, recently shown to be involved in plant cell wall biosynthesis. Lateral root primordia initiation is unaltered in this mutant. In contrast, synchronization of lateral root initiation demonstrated that mutation of *XEG113* increases the rate at which lateral root primordia develop and emerge to form lateral roots. The effect of the *XEG113* mutation was specific to the root system and had no apparent effect on shoot growth. Screening of 17 additional cell wall mutants, altering a myriad of cell wall components, revealed that many (but not all) types of cell wall defects promote lateral root formation. These results suggest that proper cell wall biosynthesis is necessary to constrain lateral root primordia emergence. While previous reports have shown that lateral root emergence is accompanied by active remodelling of cell walls overlying the primordia, this study is the first to demonstrate that alteration of the cell wall is sufficient to promote lateral root formation. Therefore, inherent cell wall properties may play a previously unappreciated role in regulation of root system architecture.

## Introduction

Post-embryonic development is the plant’s means of coping with immobility. It gives plants a way to adapt to the chaotic nature of the world around them. It also allows plants to ‘move’, slowly and methodically, towards that which they need most in life: sunlight, nutrients, and water. The latter two elements reside beneath the soil. Hence, it is the unseen organs of the root system that must respond to environmental cues to forage successfully for nutrients and water.

Hundreds or even thousands of individual roots, branched in a myriad of ways and stretching in nearly all directions, may comprise the root system of a single adult plant. However, what appears to be amazingly complex has a humble origin. For many plants, particularly those belonging to the clade eudicotyledon (including the model plant *Arabidopsis thaliana*), the complex adult root system originates from a single embryonic root. From this root develop new autonomous lateral roots. These roots give rise to more roots, and so the pattern continues. This process is neither fixed nor random, but rather highly dependent on the environment that surrounds it (Malamy, 2005, 2009; Jung and McCouch, 2013).

Within each root exists the pericycle cell layer, the outermost layer of the plant vascular bundle. The pericycle is the site of origin for new lateral roots (reviewed in De Smet, 2012). In *Arabidopsis*, the pericycle cells that border the xylem poles are the only ones that are competent to become the founders of new lateral root primordia. The first stage of primordium formation is the anticlinal division of three pairs of adjoining xylem-pole pericycle cells (Dubrovsky *et al.*, 2000; Kurup *et al.*, 2005; De Smet *et al.*, 2006; De Smet, 2012). These cells then undergo a periclinal division to double the number of cell layers in the primordia. Additional rounds of anticlinal and periclinal divisions result in a mature lateral root primordium that is ready to emerge from the confines of the parental root and begin to grow autonomously as a lateral root (Malamy and Benfey, 1997; Peret *et al.*, 2009). During the emergence process, the primordium passes through three overlying cell layers of the parental root: the endodermis, cortex, and epidermis. These cells do not disintegrate, but rather separate from each other to allow the primordium to pass between them (Swarup *et al.*, 2008). Both lateral root initiation and primordia emergence are areas of intensive study and neither is completely understood (i.e. Casimiro *et al.*, 2003; Peret *et al.*, 2009; De Smet, 2012). Both processes are regulated by a combination of intrinsic developmental programmes and responses to environmental cues, such as nutrients, light, and sucrose (Malamy, 2005, 2009; Jung and McCouch, 2013).

Recently, a novel regulator of lateral root emergence was discovered (Swarup *et al.*, 2008). *LAX3* is a high-affinity auxin importer expressed in cells overlying lateral root primordia, where its activity regulates expression of putative cell wall remodelling enzymes. Mutants in *LAX3* show reduced lateral root emergence that is correlated with a decrease in the expression of cell wall remodelling enzymes. The authors propose that the reduced expression of cell wall remodelling genes may hinder emergence by making it more difficult for cells overlying primordia to separate. Previous studies have also reported expression of putative cell wall remodelling genes around primordia and suggested that the resulting increase in cell wall remodelling proteins allows primordia to pass more easily between overlying cells (Neuteboom *et al.*, 1999; Laskowski *et al.*, 2006; Gonzalez-Carranza *et al.*, 2007). However, knockout of only one of these genes, *GLH17*, shows a (slight) alteration in lateral root emergence (Swarup *et al.*, 2008).

Here, a genetic screen of mutated *Arabidopsis* seedlings grown in culture was conducted to identify novel genes that play a role in lateral root primordia development and emergence. Mutants that showed increased lateral root formation underwent a secondary screening process to create functional subcategories of mutations. This lead to the identification of a single mutant where the increased lateral root phenotype was: (1) due to increased emergence; (2) independent of sucrose uptake from the culture media by leaves (previously shown to stimulate emergence; Macgregor *et al.*, 2008); and (3) independent of increased growth of the shoot system, which can indirectly stimulate root system development (Roycewicz and Malamy, 2012). In this mutant, named *lateral root development 5* (*lrd5*), lateral root primordia moved more quickly through all developmental stages, from initiation to emergence of the lateral root primordia from the parent root. *lrd5* has a defect in *Xyloglucotransferase113* (*XEG113*), a gene recently shown to play a role in the arabinosylation of cell wall extensins (Gille *et al.*, 2009). Examination of the roles of closely related genes, as well as unrelated genes known to be involved in various aspects of cell wall formation, revealed that many but not all alterations in cell wall formation lead to dramatic increases in lateral root formation. These findings demonstrate that lateral root primordia emergence is a default process that is constrained by properly synthesized cell walls and suggest that inherent cell wall properties may play a previously unappreciated role in regulation of root system architecture.

## Materials and methods

### Plant growth conditions

Seeds were surface sterilized in 100% bleach plus Tween-20 for 3min while vortexing and rinsed three times using autoclaved and filter-sterilized water. Seeds were imbibed for 2 or more days at 4 °C. Seeds were planted on agar media (described below) approximately 2cm from the top edge of a 100×100cm square Petri plate, with nine seeds per plate. Plates were wrapped with parafilm and placed vertically in a growth chamber with a 16/8 light/dark cycle (50–60 μmol) at 22 °C. Plants were grown for 15 d unless mentioned otherwise.

To isolate shoot tissues from contacting agar media, a strip of parafilm (about 2×9cm) was placed at the top of a Petri plate and seeds were sown just below the parafilm. Parafilm was sterilized for at least 16h in 95% ethanol.

### Media composition

Control media contained the following components (per litre): 10g sucrose, 0.5g MES, 100ml Murashige and Skoog basal salt micronutrient solution (10×, M0529; Sigma Aldrich), 5ml of 1M KNO_3_, 5ml of 1M NH_4_NO_3_, 0.33g CaCl_2_·6H_2_0, 0.1807g MgSO_4_, 0.17g KH_2_PO_4_, and 7.0g agar. Media was brought to pH 5.7 using 1M KOH prior to addition of agar, and autoclaved for 30–45min.

Other media was made by supplementing the control media with varying amounts of mannitol, sucrose, or equimolar amounts of KNO_3_ and NH_4_NO_3_. Repressive media was made by supplementing the control media with (per litre) 35g sucrose, 15ml of 1M KNO_3_, and 15ml of 1M NH_4_NO_3_.

### Determination of total lateral root length

Digital images of plant roots (taken using a Canon SD1000 digital camera) were traced by hand using ImageJ. For each seedling, the length of all lateral roots was summed to give the total lateral root length.

### Microscopic analysis of primordia initiation and emergence

Seedling roots were cleared by incubating samples sequentially in the following solutions: (1) 15min in 20% methanol acidified with 4% concentrated hydrochloric acid at 55 °C; (2) 15min in 7% NaOH in 60% ethanol at room temperature; (3) 10min in 40% ethanol at room temperature; (4) 10min in 20% ethanol at room temperature; and (5) 10min in 10% ethanol at room temperature. Glycerol (50%) was added to the last solution and stored until mounted on glass slides. Samples were observed using DIC optics on a Leica DMR microscope. Primordia were scored according to the staging system developed by Malamy and Benfey (1997).

### Plant material

Original mutant screen was performed with seeds from the Feldmann T-DNA collection (Feldmann, 1991) obtained from the *Arabidopsis* Biological Resource Center (www.arabidopsis.org). Approximately 100 pools containing 10 lines each (ABRC CS84441) were screened. Germplasm containing the *pIAA14::mIAA14-GR* construct was a gift from Hidehiro Fukaki. The following mutants were obtained from the *Arabidopsis* Biological Resource Center: *mur1-1* (CS6243), *mur2-1* (CS8565), *mur3-1* (CS8566), *mur4-1* (CS8568), *mur5-1* (CS8572), *mur6-1* (CS8573), *mur7-1* (CS8574), *mur8-1* (CS8575), *mur9-1* (CS8576), *mur10-1* (CS8577), *mur11-1* (CS8579), *prc1-1* (CS297), *ixr1-1* (CS18), *xxt1/xxt2* (CS16349), SALK_066991, SALK_053158, SALK_058092, *rra1* (CS825155), and *rra2* (CS803312).

### Determination of shoot size

Shoot size was estimated by cutting seedlings at the root–shoot junction and transferring aerial tissues to a 1.5-ml tube containing 0.5ml ethanol and incubating for 15h to extract chlorophyll. Ethanol solution (0.2ml) from each sample was transferred to 96-well plates. The absorption of the samples at 430nm was analysed using a plate reader (Tecan Safire II). To validate this method, plants were grown for 7–16 d on control media or on control media supplemented with 162mM mannitol to create plants of varying sizes. For each condition and age, 5–15 seedlings were cut at the root–shoot junction, aerial tissues were pooled, and their mass was measured. The same tissue was then extracted with ethanol and *A*_430_ and mass were compared. There was an excellent correlation between *A*_430_ and mass for plants of all ages grown under multiple conditions (Supplementary Fig. S2 available at *JXB* online). All data points represent the average on a per-plant basis of the pooled samples.

### Cloning of causal mutations

To identify the location of T-DNA insertions within the genomes of all mutants, thermal asymmetric interlaced PCR (TAIL-PCR) was performed on genomic DNA using the method and nonspecific primer sequences previously described by Liu *et al.* (1995) and primers within the T-DNA. The resulting PCR products were cloned into the vector PCR4 using the TOPO cloning system (Invitrogen) and sequenced.

### Creation of phylogeny

Phylogenetic analysis was performed using AlignX, which is part of the VectorNTI 10 software package. This program uses the neighbour-joining algorithm to construct unrooted phylogenies of the provided sequences. Amino acid sequences of LRD5 and several homologous sequences were used to create a phylogenetic tree, with a distantly related homologue from *Ostreococcus lucimarinus* as an outgroup.

### Tracking primordia emergence using *pIAA14::mIAA14-GR* construct

Seedlings containing the *pIAA14::mIAA14-GR* construct in both the *lrd5-2* and wild-type backgrounds were grown on repressive media supplemented with dexamethasone (final concentration 1 μM) for 9 d. After that time, seedlings were transferred to repressive media without dexamethasone (some seedlings were cleared and inspected to confirm that no lateral root primordia initiated in these seedlings). After transfer, seedlings were allowed to grow for 5 d. At that point, seedlings were cleared and analysed for the stage of each primordium according to the staging described by Malamy and Benfey (1997).

### Construction of GFP constructs

A PCR8 vector containing the cDNA sequence of *LRD5* was kindly provided by Sascha Gille and Markus Pauly. This vector was recombined with the vector pMDC83 (Curtis and Grossniklaus, 2003) using the Gateway LR Clonase Enzyme Mix (Invitrogen). The resulting vector was digested with *Bam*HI and *Pst*I to remove the 35S promoter, and 2kb of genomic sequence immediately upstream of the *LRD5* start site was ligated in its place. The final vector was transformed into *lrd5-2* via *Agrobacterium*-mediated transformation. Eight independent lines were selected on agar plates containing hygromycin. Of these, two lines showed strong GFP expression and only these two complemented the mutant phenotype in *lrd5-2*.

## Results

### Secondary screens identify distinct classes of lateral root mutants

Increased formation of lateral roots is a commonly reported phenotype in *Arabidopsis* mutants. Therefore, it is useful to have a pipeline to categorize mutants into subgroups representing known mechanisms for increased lateral root formation. Such a pipeline is described here for a group of eight mutants that were isolated with increased lateral root formation, and which were rescreened for lateral root emergence mutants of interest.

**Fig. 1.**
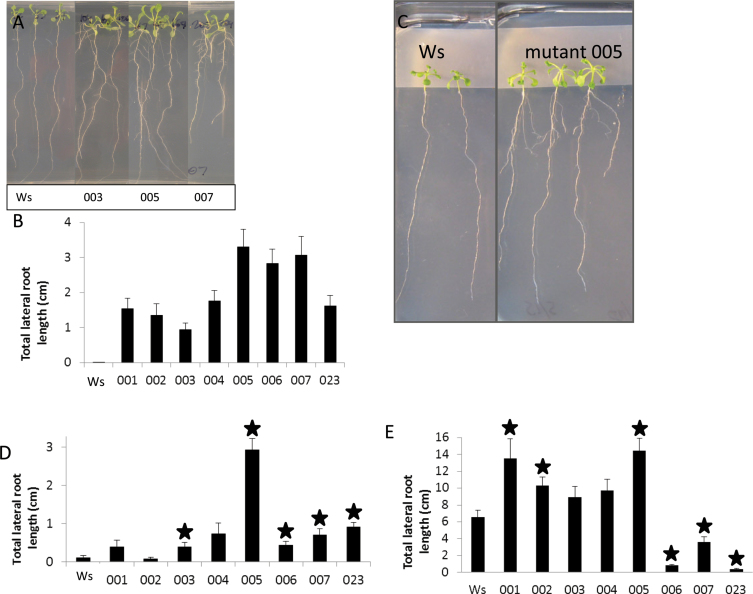
Characterization of eight mutants with increased lateral root formation compared to the wild type (Ws). (A) Three mutants showing increased lateral root formation after 15 d of growth on repressive conditions. (B) Total lateral root length for eight mutant lines compared to the wild type grown on repressive media for 15 d: all show a statistically significant increase compared to the wild type (*P*<0.01, Student’s paired t-test). (C) Mutant grown on parafilm to prevent contact between aerial tissues and media, continuing to show the lateral root phenotype. (D) Total lateral root length for eight mutant lines grown on repressive media for 18 d with aerial tissue isolated from media by parafilm; stars indicate statistically significant increases compared to the wild type (*P*<0.05, Student’s paired t-test). (E) Total lateral root length for eight mutant lines grown on control media for 14 d: mutants 001–005 show an increase in lateral root formation, mutants 006, 007 and 023 show a decrease in lateral root formation; stars indicate statistically significant differences compared to the wild type (*P*<0.05, Student’s paired t-test). Data are mean±standard error (*n*= 22–34).

The mutant screen used conditions demonstrated in Deak and Malamy (2005) and Macgregor *et al.* (2008) to strongly repress lateral root formation. For this laboratory assay, repressive conditions were defined as 1× MS basal salts with 20mM each KNO_3_ and NH_4_NO_3_, 4.5% sucrose, and low light (50–60 μmol). Seedlings grown under these repressive conditions have been previously reported to show a dramatic decrease in lateral root formation compared with seedlings grown under control conditions (control conditions were defined as 1× MS basal salts, 5mM each KNO_3_ and NH_4_NO_3_ and 1% sucrose, and low light (50–60 μmol) (Macgregor *et al.*, 2008). Seedlings from 100 pools of T-DNA mutagenized lines in the Wassilewskija (Ws) background (Feldmann, 1991; ABRC CS84441) were screened. This work identified eight mutants that showed a robust increase in lateral root formation on repressive conditions as compared to the wild type (Ws; see Fig. 1A for examples).

Lateral root formation is repressed under these screening conditions due to a number of factors. One factor is that osmotic potential affects the permeability of the aerial tissues to sucrose in the media, with lower osmotic potential reducing sucrose uptake and thereby reducing lateral root formation (Macgregor *et al.*, 2008). Another factor is osmotic repression of the overall growth rate of the plant or of the shoot system, which may exert an indirect effect on growth of the root system (Roycewicz and Malamy, 2012). Additionally, high levels of nitrate in the media have a repressive effect on lateral root formation that is independent of osmolarity, shoot growth, or shoot permeability to media sucrose (Roycewicz and Malamy, 2012). Based on this information, it is possible to predict five types of mechanisms that would allow a mutant to exhibit an increase in lateral root formation in this assay system: (1) increased permeability to media sucrose at the shoot, as has previously been shown for mutants in cuticle formation (Macgregor *et al.*, 2008); (2) constitutive increase in shoot system or whole plant growth rates; (3) reduction in nitrate-mediated repression of lateral root formation; (4) specific increase in lateral root formation independent of shoot growth; and (5) some other process is altered in a way that has yet to be uncovered. Mutants of type 4 would most likely lead to a greater understanding of the lateral root emergence process, and isolating such mutants was therefore the goal of this study.

To characterize the mutants, the average total length of all lateral roots was first determined for each line. As expected, each of the eight mutant lines identified showed a statistically significant increase in total lateral root length compared to wild type (Ws) when grown under repressive conditions for 15 d (Fig. 1A, B).

To identify type 1 mutants that overcame repression of lateral root formation via an increase in sucrose uptake by the shoot, this study investigated whether each mutant still showed a lateral root phenotype under repressive conditions with parafilm blocking aerial tissue contact with media sucrose, as in Macgregor *et al.* (2008). Five out of the eight mutants still showed a significant and reproducible increase in lateral root formation when shoots were isolated on parafilm (Fig. 1C, D), indicating that their phenotype did not depend on sucrose uptake from the media. In contrast, it is likely that mutants 001, 002, and 004 had increased lateral root formation due to increased sucrose uptake at the leaves.

Next, all eight mutants were grown under control conditions to see if any of the mutants had a growth condition-specific phenotype (i.e. ability to overcome low osmotic potential or high nitrate repression). Wild-type (Ws) seedlings grown under control conditions formed numerous lateral roots at 2 weeks of age. Interestingly, the eight mutant lines separated into two groups when grown under control conditions (Fig. 1E). Five mutants (001–005) showed an increase in lateral root formation compared to Ws under control conditions (although due to the high variability of lateral root formation on this condition, only three could be confirmed as being significantly different). Mutants 001, 002, and 004 were suggested to have increased sucrose uptake from the media under low osmotic potential (type 1), and increased lateral root formation under all conditions is consistent with that idea. Interestingly, mutants 006, 007, and 023 grown on control media showed a significant decrease in lateral root formation compared to wild type (Ws). Opposite phenotypes on the two growth conditions is not predicted for mutants of types 2, 3, or 4, and therefore these three mutants must have defects in a novel, environmentally responsive regulatory mechanism (type 5).

**Fig. 2.**
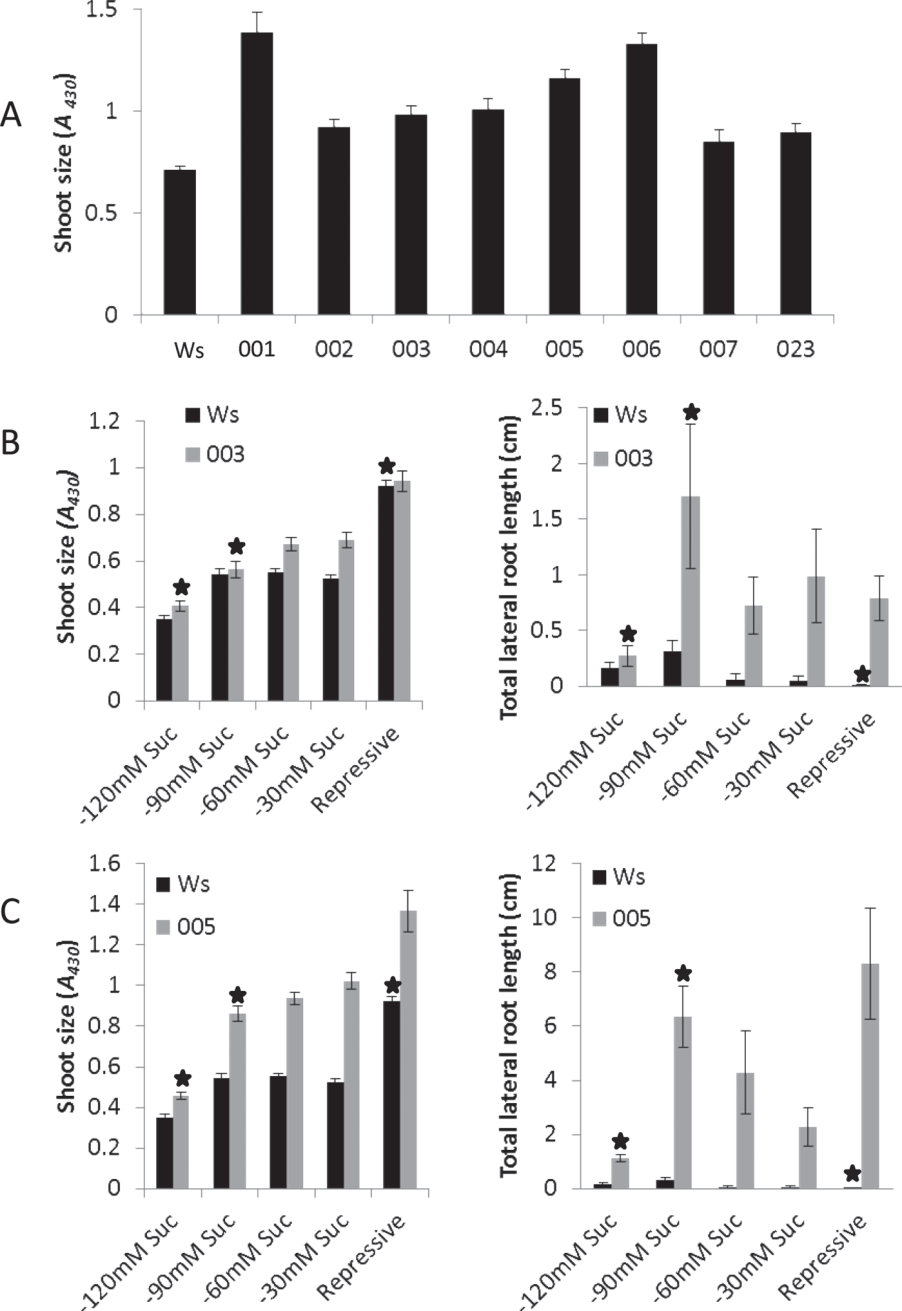
Shoot size and total lateral root length for mutants and the wild type (Ws) grown on repressive media and media with reduced sucrose and compensating mannitol content. (A) Shoot size for eight mutant lines compared to the wild type grown on repressive media for 15 d: all show a statistically significant increase compared to Ws (*P*<0.01, Student’s paired t-test). (B) Mutant 003 shows a significantly reduced shoot size (left panel) and a significantly increased total lateral root length (right panel) at 120 and 90mM sucrose when compared to the wild type (stars, *P*<0.01, Student’s paired t-test). (C) Mutant 005 shows a significantly reduced shoot size at 120mM sucrose (right panel) and significantly increased total lateral root length (left panel) (stars, *P*<0.01, Student’s paired t-test) compared to the wild type. Data are mean±standard error (*n*=16–35).

Mutants 003 and 005 became the focus of further investigation, as they had the potential to be type 4 mutants (defects specific to the lateral root formation process). To determine whether they were indeed type 4 or were exhibiting lateral root phenotypes due to increases in shoot or overall plant growth rates (type 2), shoot systems were examined. Both mutants 003 and 005 (as well as all others mutants described here) appeared to have an increased shoot size when examined visually (Fig. 1A and Supplementary Fig. S1 available at *JXB* online). To confirm this observation, seedlings were quantified for chlorophyll content using *A*_430_ as a proxy for shoot size (see Methods and Supplementary Fig. S2 available at *JXB* online). All mutants exhibited a significant increase in shoot size (Fig. 2A). To test whether root and shoot system sizes in mutants 003 and 005 are always correlated, or whether an increase in lateral root formation per unit shoot size could be found, mutants were grown on media with varying amounts of sucrose. Reducing the sucrose content in the culture media modulates the size of aerial tissues. In each medium, sucrose was replaced with an equivalent amount of the non-metabolizable sugar mannitol (to maintain a constant osmotic potential). For both mutants, reducing the concentration of sucrose resulted in seedlings with smaller or similar shoot sizes compared to Ws seedlings grown under repressive conditions (Fig. 2B, C, right panels). However, even when manipulation of the media lead to mutant plants with a smaller shoot size than Ws, the mutants still exhibited a higher total lateral root length than the Ws seedlings on repressive media (Fig. 2B, C, left panels). These results confirm that both mutants 003 and 005 are capable of forming more lateral roots per unit shoot size. Type 2 mutants are predicted to have a correlated increase in shoot and root growth, either due to an overall increase in the whole plant growth rate or an indirect effect of increased shoot growth on root growth. Thus 003 and 005 are not type 2 mutants, but rather demonstrate a root-specific phenotype, as predicted for type 4 mutants.

### Mutation in At2g35610 is responsible for lateral root phenotype in mutant 005 (*lrd5-1*)

Thermal asymmetric interlaced PCR was employed to identify the genomic location of the T-DNA insertion in mutants 003 and 005. No T-DNA was found in the 003 line either by PCR or by Southern blot analysis (not shown). However, two genomic sequences were obtained from TAIL-PCR of mutant 005, corresponding to At2g35610 and At1g20640. Segregation analysis was performed in order to determine whether either of the T-DNA insertions identified cosegregated with the mutant phenotype. The mutant was crossed to the parental background (Ws) and the resulting F1 progeny were allowed to self-fertilize. None of the F1 progeny showed a mutant phenotype, indicating that the mutation was recessive. The F2 progeny were screened for mutant phenotype, and were tested via PCR using primers specific for each T-DNA insertion (one primer internal to the T-DNA and another in the genomic DNA). Surprisingly, both T-DNA insertion sites cosegregated perfectly with the mutant phenotype.

**Fig. 3.**
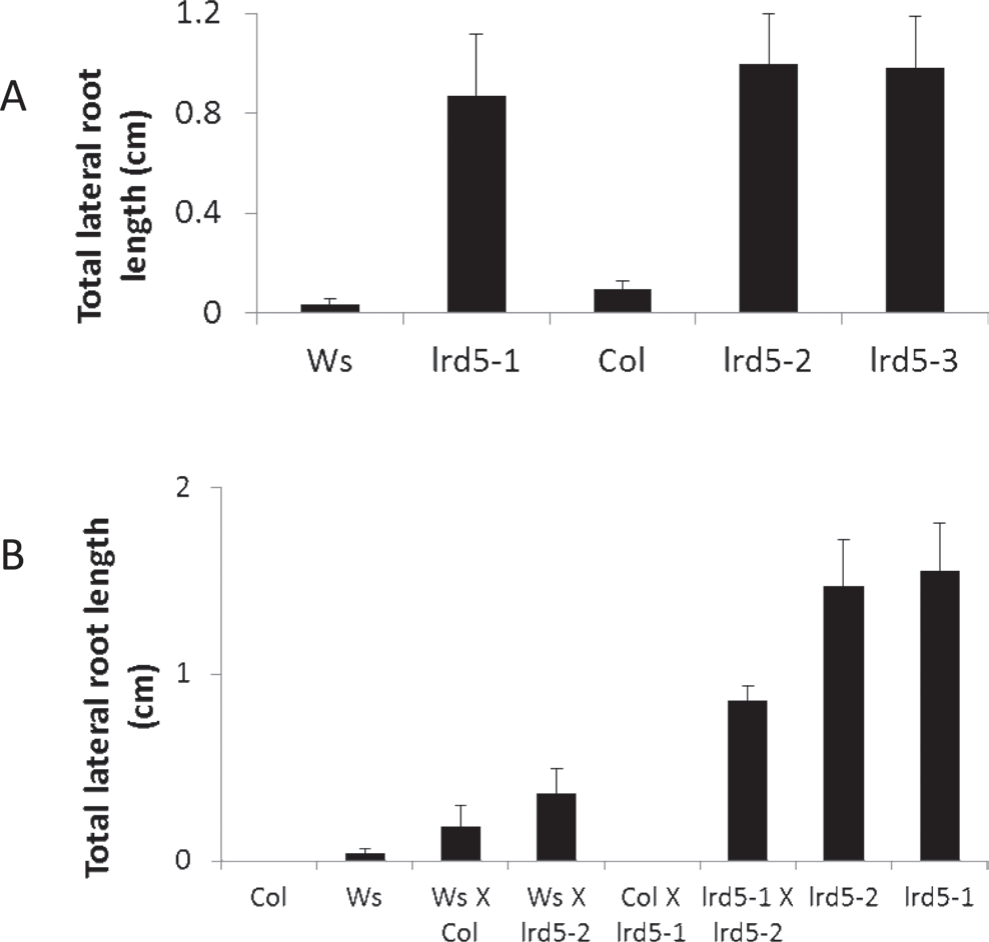
Phenotyping and complementation tests with three alleles of *LRD5*. (A) Total lateral root length for several alleles of *lrd5* grown with parafilm isolating aerial tissue from media: all mutants are significantly different from their respective wild-type backgrounds (*P*<0.01, Student’s paired t-test); data are mean±standard error (*n*=23–63); *lrd5-1* is in the Ws background while *lrd5-*2 and *lrd5-3* are in the Col background. (B) Total lateral root length for the wild type, two alleles of *lrd5*, F1 plants from complementation test crosses, and F1 plants from control crosses; data are mean±standard error (*n*=17–53). Differences between *lrd5-1*×*lrd5-2* and all controls are significant (*P*<0.01, Student’s paired t-test).

Mutant 005 was designated *lateral root development 5 (lrd5-1)*. Of the two T-DNA insertions identified in *lrd5-1*, the first was located within an intron in gene At2g35610 and the second within an exon in gene At3g20640. Since the T-DNA insertions were predicted to be on separate chromosomes, the fact that both T-DNA insertions cosegregated perfectly with each other and with the mutant phenotype suggests the possibility of genomic rearrangement in *lrd5-1*. To determine which mutation was responsible for the mutant phenotype, SALK T-DNA alleles of At2g35610 SALK_066991 (*lrd5-2*) and SALK_058092 (*lrd5-3*) were examined (no alleles of At3g20640 were available at the time of this work). These alleles are in the Col background. Both *lrd5-*2 and *lrd5-*3 showed increased total lateral root length, like the original mutant *lrd5-1*, when grown with parafilm blocking contact between the aerial tissues and media (Fig. 3A). Additionally, a cross between the original T-DNA mutant *lrd5-1* and *lrd5-2* failed to rescue the mutant phenotype (Fig. 3B; since the two alleles are in different backgrounds, crosses are included to eliminate the possibility of interactions between mutant alleles and disparate genetic backgrounds) These results confirm that the T-DNA insertion in At2g35610 is causal for the mutant phenotype in *lrd5-1*.

### *LRD5* modifies cell wall proteins

**Fig. 4.**
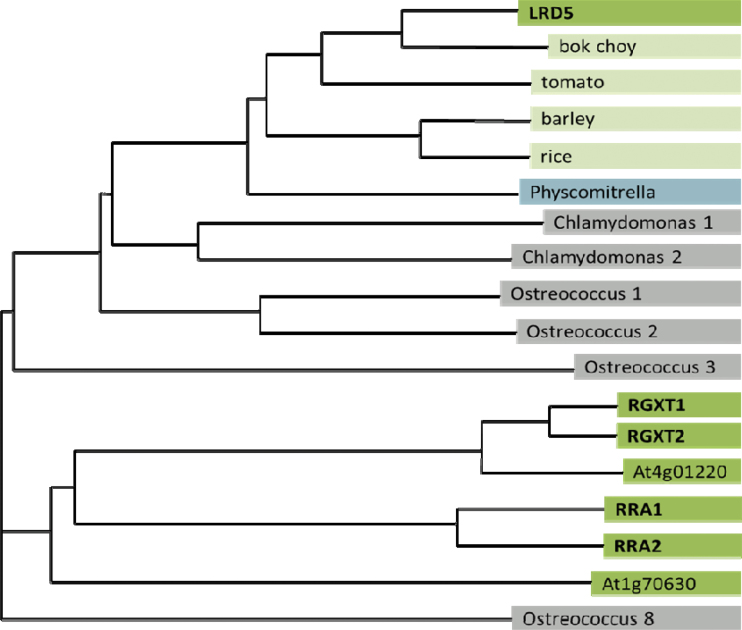
Amino acid phylogeny using the neighbour-joining method using select sequences identified from a BLAST search using the protein sequence of LRD5. Several species included several homologous sequences, which are individually numbered. RGXT1, RGXT2, RRA1, and RRA2 have all been shown to be involved in cell wall biosynthesis.

*LRD5* encodes a gene with no known domains or motifs. Previous computational studies have grouped *LRD5* within the glycosyltransferase family 77 based on homology to other glycosyltransferases (www.cazy.org). This result was confirmed by a BLAST search using the predicted protein sequence of *LRD5* against the NCBI sequence database. Six *Arabidopsis* genes showed significant homology to *LRD5* (Fig. 4), all of which belong to the glycosyltransferase family 77 (www.cazy.org). Several of these genes have been previously identified to play a role in cell wall biosynthesis (Egelund *et al.*, 2006, 2007, 2008). The most closely related gene, At1g70630, is predicted to encode a protein with 51% amino acid similarity over a stretch of 155 amino acids located towards the middle of the protein sequence. However, no significant similarity was found along the rest of the protein sequence. This gene was recently named Reduced Arabinose Yariv1 (RAY1) and shown to play a role in arabinosylation of cell wall arabinogalactan proteins (AGPs; Gille *et al.*, 2013).

The BLAST search also identified sequences in over 30 plants, the moss *Physcomitrella patens*, and three algae (*O. lucimarinus*, *Ostreococcus taurii*, and *Chlamydomonas reinhardtii*) that showed greater homology to *LRD5* than the closest homologue in *Arabidopsis* (selected examples shown in Fig. 4). Given that *Arabidopsis* diverged from *Ostreococcus* and *Chlamydomonas* roughly 1 billion years ago (Yoon *et al.*, 2004), this suggests that *LRD5* diverged from its closest *Arabidopsis* homologue at least that long ago.

Recently, mutants in *LRD5* (*lrd5-2/xeg113-2* and *lrd5-3/xeg113-3*) were characterized and shown to have an increase in etiolated hypocotyl length, rosette size, and early inflorescence bolting (Gille *et al.*, 2009) and root hair growth (Velasquez *et al.*, 2011). These mutants contained reduced arabinosylation of cell wall extensins and the authors proposed that *LRD5* functions as an extensin arabinosyltransferase. The authors concluded that extensin glycosylation by *At2g35610* is an important determinant for proper cell elongation. However, it was not immediately obvious how these results relate to the lateral root phenotype seen in *lrd5* mutants.

### Mutants in *LRD5/XEG113* show an increased rate of lateral root development and emergence

**Fig. 5.**
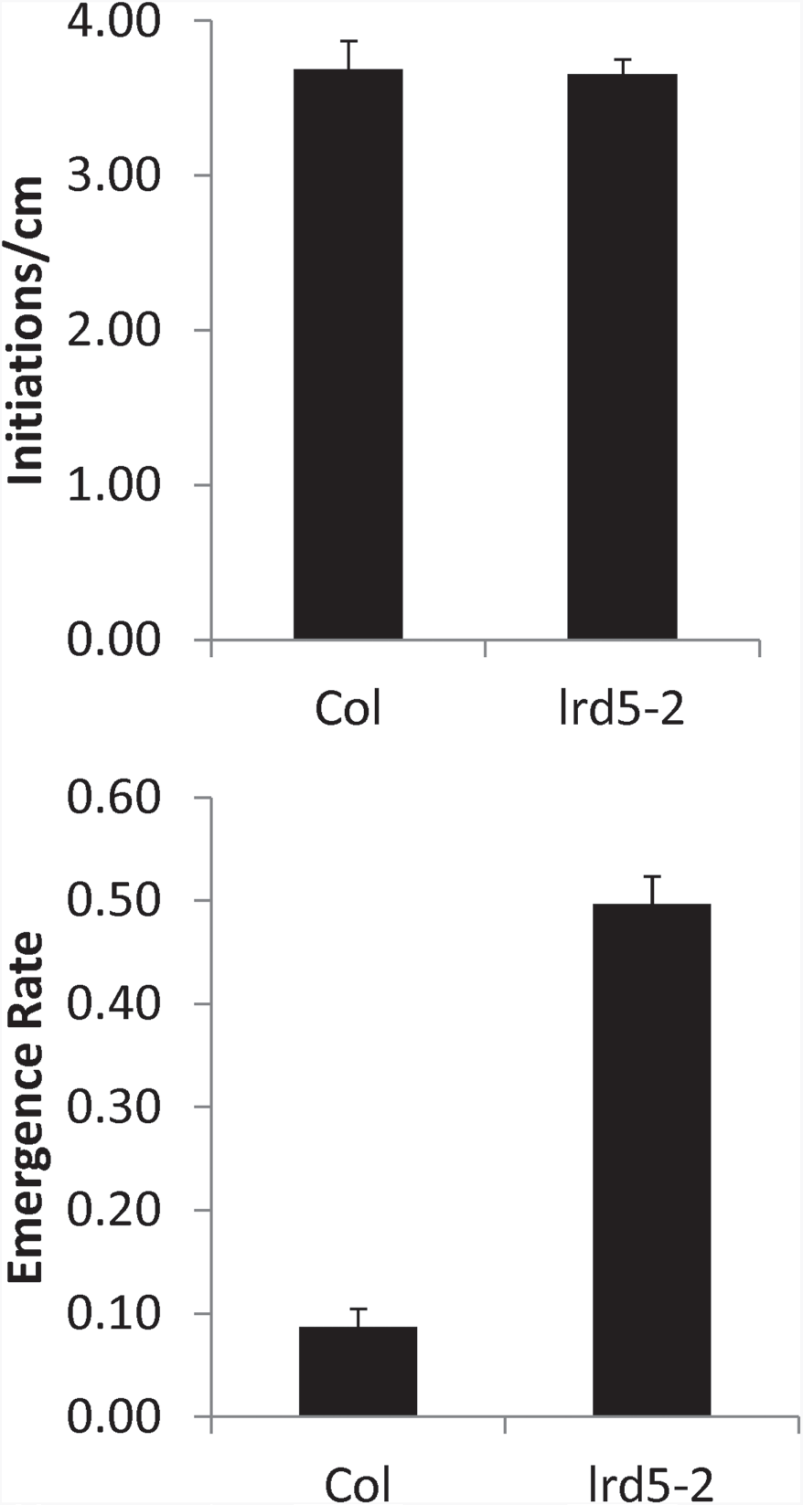
Number of initiations and emergence rate for *lrd5-2* and the wild type (Col). Emergence rate is measured as the number of emerged primordia divided by total number of primordia. A total of 161 primordia were analysed for *lrd5-2* and 254 for Col. No difference in initiations per cm was seen in the mutant (Student’s paired t-test). In contrast, emergence rate was significantly increased (*P*<0.01, pooled z-statistic). Data are mean±standard error (161 primordia in 10 seedlings *lrd5-2* and 254 primordia in 10 seedlings for Col).

The increase in lateral root formation observed in mutants in *LRD5/XEG113* may be due to an increase in the number of initiated primordia, the rate at which primordia emerge, or both. This question was addressed by analysing the density of initiations and the frequency of emergence in 14-day-old *lrd5-2* and wild-type (Col) seedlings. *lrd5-2* seedlings showed no increase in the number of primordia, but a large increase in the frequency with which these primordia emerge (Fig. 5). (A striking increase in emergence was also seen in *lrd5-1* in comparison to its wild-type background (Ws) (Supplementary Fig. S3A available at *JXB* online), although the phenotype was less pronounced due to the higher emergence rate in Ws vs. Col). These results suggest that aberration in some aspect of the emergence process strongly contributes to the lateral root formation phenotype in *lrd5* mutants, although the possibility remains that an increase in lateral root growth rate also contributes to the phenotype.

**Fig. 6.**
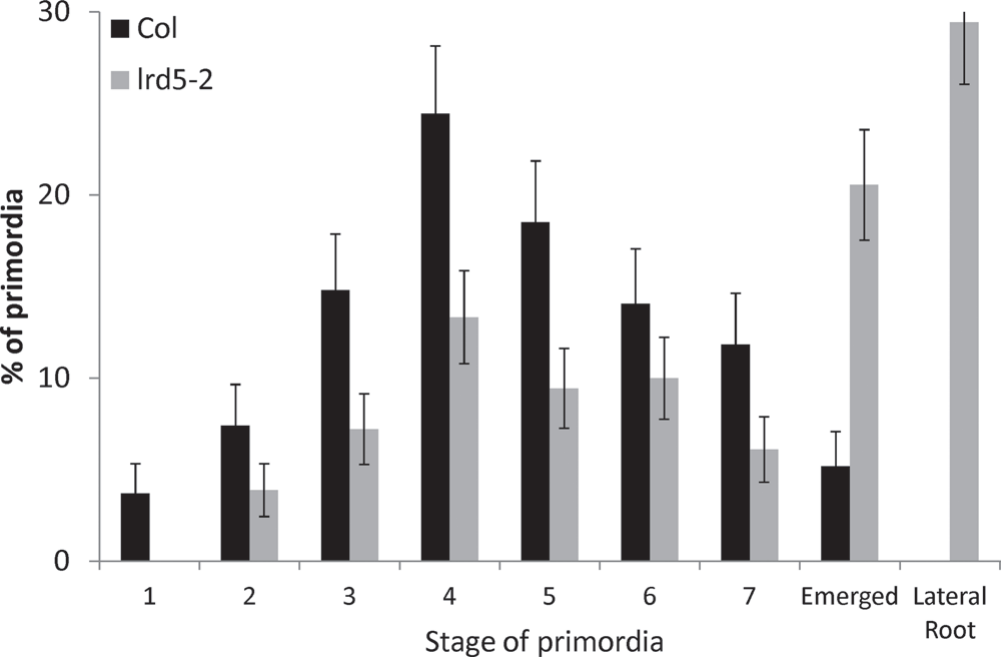
Proportion of lateral root primordia at each stage 5 d after release of SLR1 inhibition of initiation in *lrd5-2* and wild type (Col). All comparisons except stage 6 are statistically significant (*P*<0.05, pooled z-statistic. Data are mean±standard error (*n*=16).

Given that primordia emerge more frequently in *lrd5*, the developmental stages of the primordia were next examined. To more accurately monitor the developmental process of lateral root primordia in wild-type and *lrd5* seedlings and to allow a detailed comparative time course of lateral root development, lateral root initiation was synchronized. *lrd5-2* was crossed into plants containing the *pIAA14::mIAA14-GR*, a dexamethasone-inducible dominant allele of *IAA14* (Fukaki *et al.*, 2005). *IAA14* (*SLR1*) is a major component of auxin-mediated lateral root initiation, and a dominant mutation of *IAA14*, *mIAA14*, completely abolishes lateral root initiation. Grown in the presence of dexamethasone, *pIAA14::mIAA14-GR* seedlings do not initiate any primordia; upon removal of dexamethasone, *pIAA14::mIAA14-GR* seedlings initiate primordia in young tissues. This system thus allows for the synchronous initiation of primordia in seedlings old enough to form lateral roots. Wild-type (Col) and *lrd5-2* seedlings containing the *pIAA14::mIAA14-GR* construct were grown on repressive media containing dexamethasone for 9 d and then transferred to media lacking dexamethasone. After 5 d, the seedlings were cleared and primordia were scored based on the staging described by Malamy and Benfey (1997) (Fig. 6). *lrd5-2*/*pIAA14::mIAA14-GR* seedlings contained fewer primordia than the wild type at each of the seven pre-emergence stages, but more emerged primordia. Since no differences in initiation rate are predicted in *lrd5-2*, this result must be explained by an increase in the rate at which primordia progress through the entire developmental process. The reduction even in stage 1 and 2 primordia indicates that the increased rate of development starts around the time that primordia first initiate.

### *LRD5/XEG113* does not directly affect whole-plant growth

**Fig. 7.**
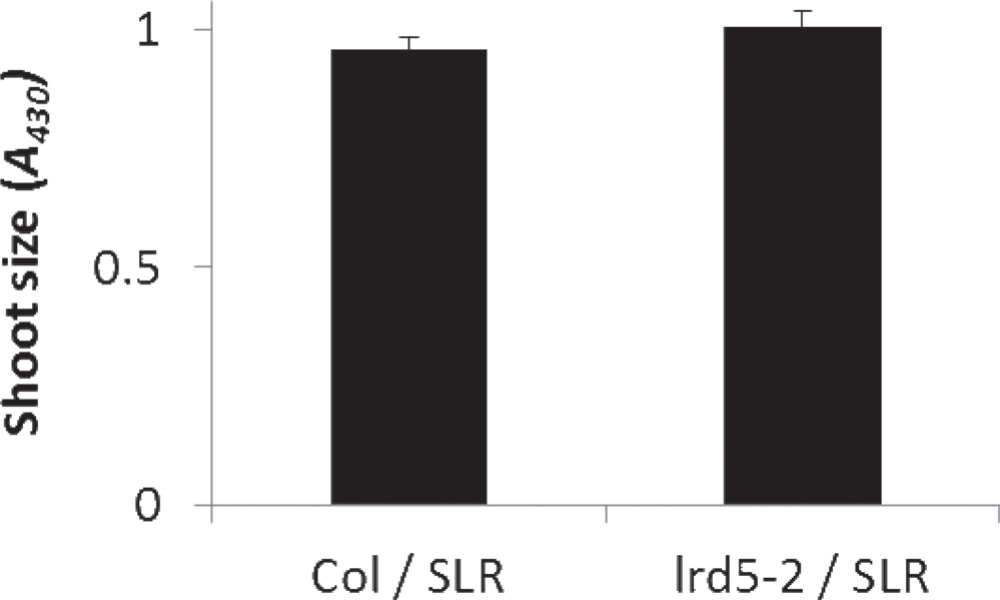
Aerial tissue size (*A*_430_) of the wild type and *lrd5-2* with *mIAA14* induced by dexamethasone. Differences between the two genotypes are not statistically significant (Student’s paired t-test). Data are mean±standard error (*n*=39).

The increase in lateral root formation per unit shoot size (Fig. 2) suggests that the effects of the *LRD5/XEG113* mutation are root specific. This would further predict that the increase in aerial tissue growth observed here (Fig. 1) and by Gille *et al.* (2009) in *lrd5/xeg113* mutants is a result of the increase in lateral root growth rates. To test this idea, wild-type (Col) and *lrd5-2* seedlings containing the *pIAA14::mIAA14-GR* construct were grown on media with dexamethasone for 14 d. Indeed, when lateral root formation was inhibited in *lrd5-2/xeg113*, shoot size was indistinguishable from the wild type (Fig. 7). This indicates that changes in shoot growth in *lrd5* are secondary, and not causal, to increased lateral root formation.

### *LRD5/XEG113* is expressed in trichoblasts and cells near the root tip

**Fig. 8.**
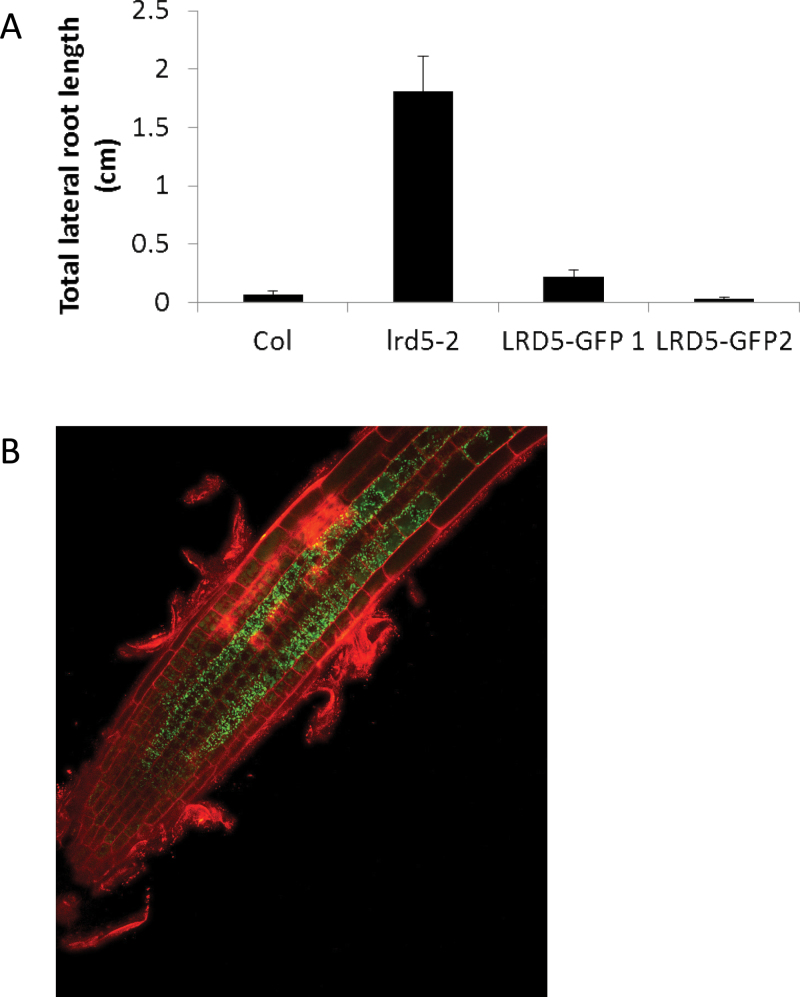
Endogenous expression pattern of *LRD5*. (A) Total lateral root length for *lrd5-2* and *lrd5-*2 with two separate rescue constructs. *lrd5-2* is significantly different from all other genotypes (*P*<0.01, Student’s paired t-test). Data are mean±standard error (*n*=47–77). (B) Strong GFP expression is seen in the trichoblast cell file in plants transformed with constructs shown in A.

To better understand how *XEG113*/*LRD5* functions in the root to affect lateral root development, a transgenic construct consisting of 2kb promoter sequence driving the wild-type cDNA fused to GFP (*pLRD5::LRD5-GFP*) was created. This construct was transformed into *lrd5-2* and two independent lines were identified that showed strong GFP expression and also rescued the mutant lateral root phenotype by reducing total lateral root length (Fig. 8A) and lateral root emergence rates (Supplementary Fig. S3B available at *JXB* online).

*LRD5/XEG113* is most strongly expressed in the trichoblast cell file just basal to the primary root tip, with decreasing expression intensity in more mature regions of the root (Fig. 8B). These results are consistent with the publicly available microarray data (www.arexdb.org). The microarray data also suggests that *LRD5* is expressed at low levels in most cell layers just behind the primary root tip. This expression is barely visible using fluorescence microscopy (not shown). No expression is predicted or can be seen in lateral root primordia or cells overlying primordia (not shown).

The finding that *LRD5/XEG113* is expressed near the root tip and not expressed in or around primordia suggests that this gene either acts non-cell autonomously or plays a role in early wall formation in cells that will later play a role in primordia emergence (i.e. cells that will come to overly a primordium later in development). Since it is difficult to imagine how trichoblast cell wall composition would affect lateral root primordia emergence, it is hypothesized that the second model is correct (see Discussion).

### Related and unrelated cell wall mutants also show a lateral root phenotype

**Fig. 9.**
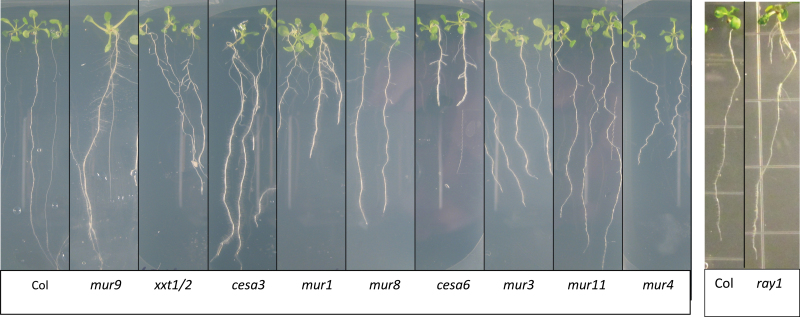
Phenotypes of cell wall mutants grown for 14 d on repressive conditions. Many cell wall mutants show lateral root phenotypes.

Mutations in *LRD5/XEG113* result in reduced arabinosylation of extensins in the cell wall (Gille *et al.*, 2009) and increased rate of lateral root development and emergence (this study). To test whether other Family 77 glycosyltransferases showed lateral root phenotypes under the screening condition, knockout lines were obtained for At1g70630 (*RAY1*, the closest homologue to *XGE113/LRD5* in *Arabidopsis*), *RRA1* and *RRA2*. The latter two mutants, although altered in closely related and tandemly duplicated genes, show slight reductions in arabinose content (Egelund *et al.*, 2007). (Mutants in *RGXT1*, *RGXT2*, and *RGXT3*, previously studied Family 77 genes, were not tested as *RGXT1* and *RGXT2* are tandemly duplicated genes with no detectible phenotypes/defects when mutated and confirmed alleles of *RGXT3* are not available). Whereas *rra1* and *rra2* had no detectible lateral root phenotype, the mutant allele of At1g70630 (Salk_053158, *ray1*) showed a dramatic increase in lateral root formation compared to the wild type (Col) (Table 1 and Fig. 9). To see if At2g35610 and At1g70630 act in parallel, *lrd5-2/xeg113* and *ray1* were crossed to form a double mutant. Although the double mutant did not have altered lateral root formation compared to *ray1* (the mutant with most lateral root formation) with aerial tissues contacting media, on parafilm the double mutant showed a much stronger phenotype compared to both *lrd5-2* and *ray1* (Table 1). *RAY1* was recently shown to play a role in arabinosylation of AGPs and not extensins (Gille *et al.*, 2013). This suggests that two Family 77 glycosidases with varying functions in cell wall modification affect lateral root formation and that the *lrd5/xeg113* phenotype is not specific to extensin modification.

**Table 1.** Total lateral root length for cell wall mutants grown with and without parafilm to isolate shoots from mediaValues are mean±standard error. Asterisks indicate statistically significant deviations from wild type (*P*<0.05 using Student’s paired t-test). Total lateral root length for *lrd5-2* and *ray1* are significantly different from the *lrd5-2/ray1* double mutant. AGP, arabinogalactan protein; NK, not known.

	Perturbed in:		Total lateral root length (cm)	
	Gene	Cell wall	Off parafilm	On parafilm
Col	NK	NK	0.18±0.08	0.10±0.05
Cell wall mutants				
*mur1* ^*a*^	At3g51160	Fucose	2.27±0.24*	1.65±0.26*
*mur2* ^*a*^	At2g03220	Fucose	0.01±0.00	–
*mur3* ^*a*^	At2g20370	Fucose	0.41±0.16*	0.24±0.15
*mur4* ^*a*^	At1g30620	Arabinose	0.86±0.12*	2.40±0.35*
*mur5* ^*a*^	NK	Arabinose	0.13±0.05	–
*mur6* ^*a*^	NK	Arabinose	0.09±0.03	–
*mur7* ^*a*^	NK	Arabinose	0.05±0.02	–
*mur8* ^*a*^	NK	Rhamnose	1.23±0.19*	0.73±0.34*
*mur9* ^*a*^	NK	Fucose, xylose	2.94±0.28*	0.09±0.04
*mur10* ^*a*^	At5g17420	Cellulose, fucose, arabinose, xylose	0.11±0.03	–
*mur11* ^*a*^,^*b*^	At3g59770	Rhamnose, fucose, xylose, mannose	0.47±0.10*	0.70±0.30
*Cesa6* ^*c*^	At5g64740	Cellulose	1.28±0.11*	3.04±0.29*
*cesa8* ^*d*^	At4g18780	Cellulose	2.73±0.24*	3.51±0.32*
*xxt1/xxt2* ^*e*^	At4g02500/At3g62720	Xyloglucan	4.13±0.36	2.43±0.58
Family 77 GTs				
*lrd5-2*	At2g35610	Extensin arabinose	0.77±0.11*	0.80±0.22*
*rra1* ^*f*^	At1g75120	Arabinose	0.10±0.04	–
*rra2* ^*f*^	At1g75110	Arabinose	0.07±0.03	–
*ray1* ^*g*^	At1g70630	AGP arabinose	5.21±0.54*	0.85±0.18*
*lrd5-2/ray1*	At2g35610/At1g70630	–	5.16±0.42*	2.12±0.28*

a Reiter *et al.*, 1997; ^b^ Austin et al., 2011;^c^ Turner and Somerville, 1997; ^d^ Fagard *et al.*, 2000; ^e^ Cavalier *et al.*, 2008; ^f^ Egelund *et al.*, 2007; ^g^ Gille *et al.*, 2013

To further investigate the relationship between cell wall properties and lateral root formation, a panel of mutants previously characterized to have altered cell wall composition were screened for lateral root phenotypes (Fig. 9). Attention was focused on the *murus* (*mur*) series of mutants, as well as *prc1* (*cesa6*), *irx1* (*cesa8*), and *xxt1*/*xxt2* (Reiter *et al.*, 1997; Turner and Somerville, 1997; Fagard *et al.*, 2000; Cavalier *et al.*, 2008). This set includes mutants with altered levels of cellulose, xyloglucan, fucose, arabinose, rhamnose, xylose, and/or mannose in the cell wall (all but one of the mutants altered in arabinose have not been cloned and therefore it is unknown if they are altered in the same pathway as *lrd5*). Interestingly, nine out of 14 mutants in this set showed an increase in lateral root formation compared to the wild-type background (Col) when grown under repressive conditions (Table 1). Lateral root formation was also analyzed in each of the cell wall mutants grown on parafilm to restrict contact between the leaves and the nutrient media, to reveal whether differences in shoot uptake of media sucrose could account for the root system phenotypes, as has been seen previously for mutants in cuticle formation (Macgregor *et al.*, 2008). While the phenotypes of *mur3*, *mur9*, and *mur 11* might be attributable to this phenomenon, the remaining mutants tested still showed increased lateral root formation when their shoots were isolated on parafilm, indicating that these mutants form increased numbers of lateral roots independent of nutrient uptake through aerial tissues. Together, these results demonstrate that the phenotype seen in *lrd5/xeg113* is not specific to cell wall extensin arabinosylation or to cell wall arabinose levels, but is shared with many mutants that compromise cell wall biosynthesis.

## Discussion

### A pipeline for categorizing mutations with direct and indirect effects on lateral root formation

Lateral root formation can be broadly considered to involve lateral root initiation, primordia development and patterning, and primordia emergence. Mutations in any of these processes (and others) manifest as altered numbers of lateral roots. This study demonstrates a pipeline that distinguishes between the mechanisms compromised in a set of eight mutants with increased lateral root formation. Such a pipeline should be broadly useful for understanding whether lateral root mutant phenotypes are an indirect result of altered plant/shoot system growth, are a result of shoot tissues that are ‘leaky’ to sucrose in culture media, or can truly be described as identifying genes involved in root development. It allowed the identification of *lrd5/xeg113* as a mutant specifically affected in lateral root development and emergence.

### The role of *LRD5/XEG113* in constraining lateral primordium emergence

Gille *et al.* (2009) demonstrated that *LRD5/XEG113* is involved in elongation of arabinoside chains on extensins. Extensins are hydroxyproline-rich glycoproteins that are embedded in the plant cell wall, and it is believed that their role is strictly to provide mechanical reinforcement (Lamport, 2001). This suggests that the effect of *LRD5/XEG113* on lateral root formation is mediated by its role in the physical characteristics of the cell walls. Given that extensins are thought to strengthen the cell wall, one possibility is that cell wall modifications catalysed by *LRD5/XEG113* make it more difficult for primordia to push between overlying cell layers during emergence. More recent work has shown that XEG113 and other enzymes involved in extensin modification play an important role in polarized cell growth in root hairs and pollen tubes (Velasquez *et al.*, 2011; Ogawa-Ohnishi *et al.*, 2013). However, the idea that *LRD5/XEG113* contributes to a broad characteristic of the cell wall, such as strength, is more consistent with the fact that many unrelated mutants in cell wall synthesis demonstrate similar lateral root phenotypes. This makes it unlikely that the *lrd5* phenotype is specifically associated with modification of extensins or arabinose chain length.

Transgenic plants expressing *LRD5/XEG113:GFP* fusions under the control of an endogenous promoter suggest that, in the root, *LRD5/XEG113* is expressed most strongly in trichoblasts near the root tip. This has not been previously demonstrated, but is strongly predicted by microarray experiments (http://www.arexdb.org). Furthermore, trichoblast expression is consistent with the demonstrated important role of XEG113 in root hair growth (Velasquez *et al.*, 2011). Therefore, the reporter lines are likely to accurately reflect *LRD5/XEG113* expression. It seems unlikely that the loss of LRD5/XEG113 from trichoblasts is responsible for increased lateral root primordia emergence in the *lrd5* mutants. Instead, it is hypothesized that low levels of expression in other cell types at the root tip, also predicted by microarray, are responsible. In this model, critical cell wall characteristics are established in young cells that will come to overlay lateral root primordia later in development. Rescue of the *lrd5* phenotype with tissue-specific expression will be necessary to test this model. However, the preferential expression of *LRD5/XEG113* in young trichoblast cells, long before root hair formation begins, suggests that the early events in cell wall formation can be manifested later in development.

### The role of the cell wall in constraining lateral primordium emergence

Some cell wall mutants affected lateral root primordia emergence while others did not. The set that showed a lateral root phenotype included *prc1* and *ixr1* (altered in cellulose deposition), *xxt1/xxt2* (no detectible xyloglucan), *mur1* (reduced fucose in xyloglucan, rhamnogalacturonan II, and arabinogalactan proteins), *mur3* (reduced fucose in xyloglucan), *mur4* (almost no arabinose in rhamnogalacturonan I and II, glucoarabinoxylans, hydroxyproline-rich proteins, and arabinogalactan proteins), and *mur8*, *mur9*, and *mur11* (three mutants altered in cell wall rhamnose, fucose, xylose, and mannose content) (Bonin *et al.*, 1997; Reiter *et al.*, 1997; Turner and Somerville, 1997; Fagard *et al.*, 2000; Vanzin *et al.*, 2002; Burget *et al.*, 2003; Bosca *et al.*, 2006; Cavalier *et al.*, 2008). Only two of these mutants had been previously reported to have lateral root phenotypes (van Hengel and Roberts, 2002; Hermans, 2010). This data raises the question of what these mutants have in common that distinguishes them from cell wall mutants that fail to show a lateral root phenotype. It is possible that genes affecting lateral root formation act to strengthen the walls of cells overlying primordia, as hypothesized here for *LRD5/XEG113*. All of the genes perturbed in the tested mutants that have altered lateral root emergence (and have been cloned) are predicted to be expressed in cells that at some point overly primordia (www.arexdb.org). *mur10*, on the contrary, is not expressed in cells overlying primordia and does not have a lateral root phenotype. Although not conclusive, this observation is consistent with the hypothesis that alterations in the cells that overly primordia are responsible for the emergence phenotype seen in the mutants. Mechanical tests on hypocotyls in *mur1*, *mur3*, *mur10*, and *xxt1*/*xxt2* have shown that these mutants have altered cell wall strength (Ryden *et al.*, 2003; Pena *et al.*, 2004; Bosca *et al.*, 2006; Cavalier *et al.*, 2008; Abasolo *et al.*, 2009). In contrast, *mur2*, although altered in the fucosylation of xyloglucan along with *mur1* and *mur3*, is only marginally weaker in mechanical tests compared to the wild type (Ryden *et al.*, 2003; Pena *et al.*, 2004; Abasolo *et al.*, 2009) and does not have a lateral root phenotype. This may imply that the reduction in strength in *mur2* is insufficient to cause an alteration in lateral root emergence. Despite these correlations, it remains possible that a broad change in cell wall composition is shared by mutants with lateral root phenotypes and that this change affects lateral root formation by a mechanism independent of cell wall strength. Indeed, in the *lrd5* mutant, all stages of lateral root development were accelerated (Fig. 6). This might be evidence that cell walls play a role in growth and development that is more complex than creating physical constraint.

The notion that the walls of cells overlying primordia are important in restricting emergence has been proposed previously in the literature (Neuteboom *et al.*, 1999; Laskowski *et al.*, 2006; Gonzalez-Carranza *et al.*, 2007; Swarup *et al.*, 2008; Peret *et al.*, 2009). A similar role was recently shown for the root casparian strip, which apparently constrains the transition of the lateral root primordia from a flat to rounded morphology during development (Lucas *et al.*, 2013). However, the present work is the first, as far as is known, to suggest that a weakening or modification of the cell wall is sufficient to promote lateral root primordia emergence. This would mean that lateral root primordia are poised for emergence and are constrained merely by the presence of overlying layers of cell walls. This is consistent with the model of Peret *et al.* (2009), who proposed that, during normal development, LAX3 expression in cells overlying the primordia allows auxin to induce cell wall remodelling enzymes in these cells, leading to cell separation and lateral root primordia emergence. This is also consistent with the observations of Lucas *et al.* (2013), who showed that expression of a dominant negative *AXR3* allele in tissues overlying a primordium delayed development, which they interpret to mean that primordia cannot emerge in the absence of auxin-induced cell wall remodelling.

This study suggests that mutations, environmental response pathways, or intrinsic pathways that weaken or otherwise alter cell walls may be sufficient to promote lateral root formation by facilitating the separation of the cells overlying the lateral root primordium. One way to test this model further would be to introduce the *lrd5/xeg113* or *ray1* mutations into a *lax3 aux1* double-mutant background to see if cell wall defects in *lrd5* or *ray1* could compensate for the putative loss of a mechanism to soften cell walls. However, triple mutant progeny could not be recovered from either *aux1/aux1 lax3/lax3 lrd5/+* or *ray1/+* lines. This suggests a strong interaction between auxin import and cell wall composition that will require further work to decipher.

## Supplementary material

Supplementary data are available at *JXB* online.

Supplementary Fig. S1. Shoot systems of eight mutants isolated based on increased lateral root formation.

Supplementary Fig. S2. Correlation of plant shoot mass and absorbance of ethanol extracts.

Supplementary Fig. S3. Lateral root emergence in a second *lrd5* allele and after rescue.

Supplementary Data
