# Supplementary material for: Cell wall properties play an important role in the emergence of lateral root primordia from the parent root
Source: J Exp Bot. 2014 Mar 11;65(8):2057–69. doi: 10.1093/jxb/eru056 (PMC3991740; doi:10.1093/jxb/eru056)
Supplement: Supplementary Data [file supp_65_8_2057__index.html]

Cell wall properties play an important role in the emergence of lateral root primordia from the parent root — Cell wall properties play an important role in the emergence of lateral root primordia from the parent root — Supplementary Data 

# Cell wall properties play an important role in the emergence of lateral root primordia from the parent root

## Supplementary Data

Data files

**Files in this Data Supplement:**

- Supplementary Data - Supplementary Data
