# Supplementary material for: Cell wall properties play an important role in the emergence of lateral root primordia from the parent root
Source: J Exp Bot. 2014 Mar 11;65(8):2057–69. doi: 10.1093/jxb/eru056 (PMC3991740; doi:10.1093/jxb/eru056)
Supplement: Supplementary Data [file supp_eru056_jexbot108860_file001.pdf]

Figure S1. Shoot systems of eight mutants isolated based on increased lateral root formation. Bar = 0.5 cM.

Figure S1

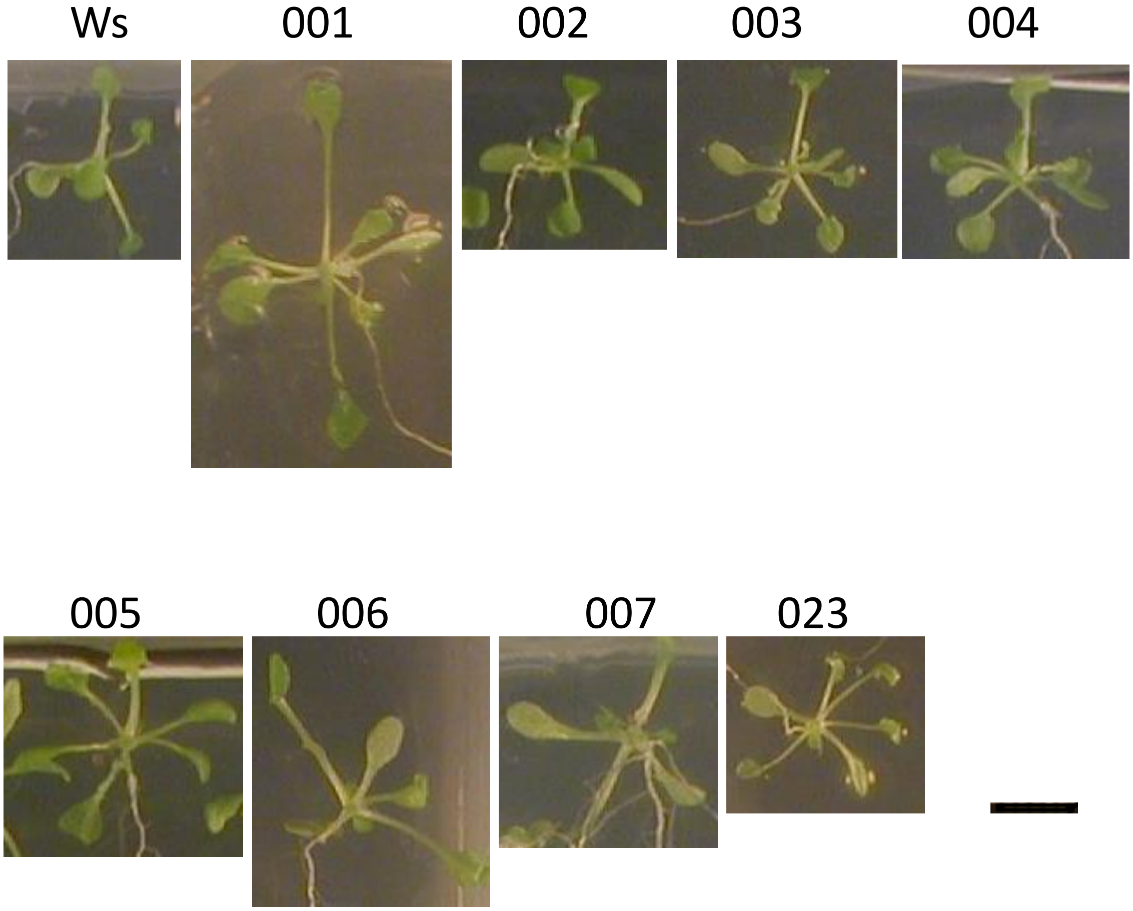

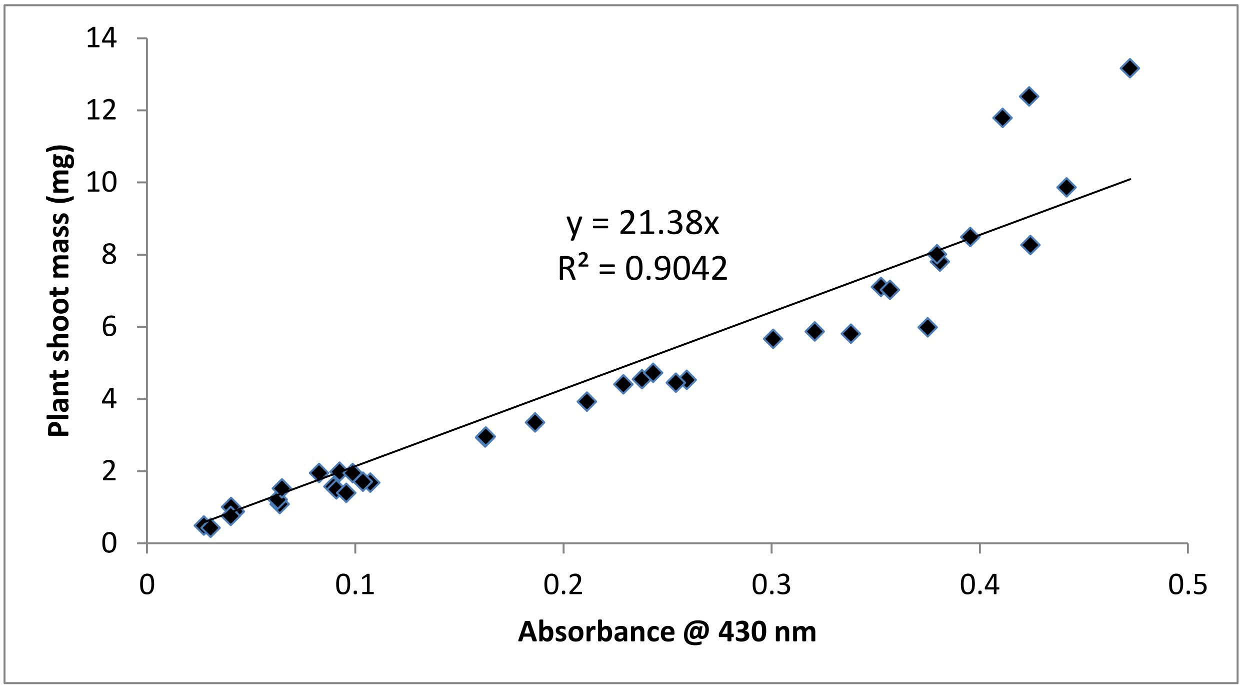

Figure S2. Correlation of plant shoot mass and absorbance of ethanol extracts.

Figure S3 Lateral root emergence in a second *lrd5* allele and after rescue.

A. Lateral root emergence in *lrd5-1.* Emergence is calculated as number of emerged lateral roots per total initiation events. The *lrd5-1* mutant shows increased lateral root emergence under repressive conditions (Students T-test, P=.008). B. Lateral root emergence is rescued by the LRD5:GFP construct described in the text (lrd5R), confirming a role for this gene in repression of emergence .

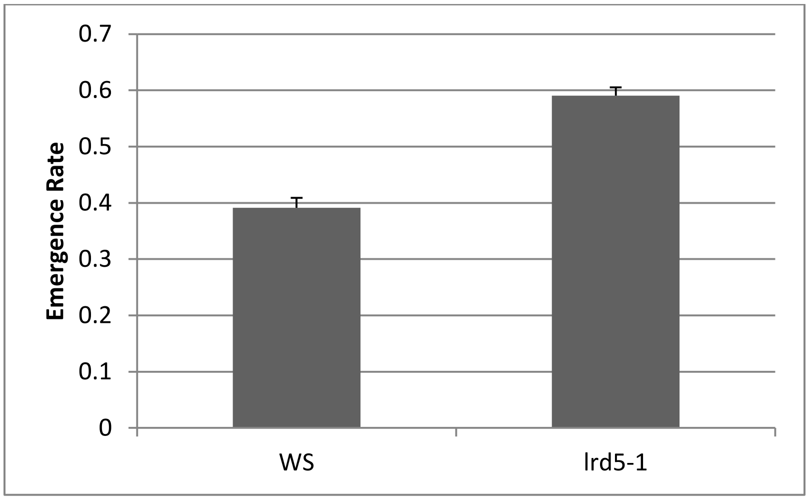

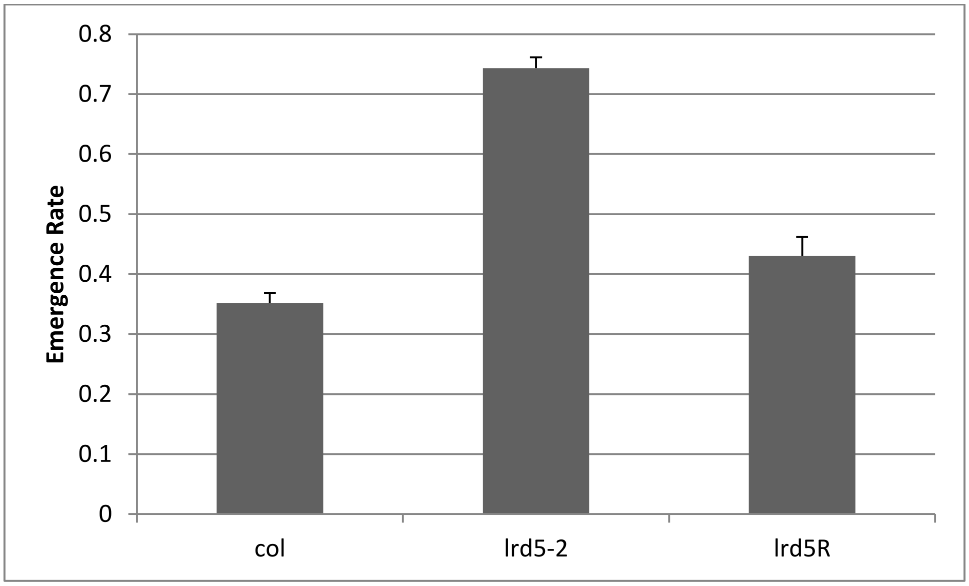

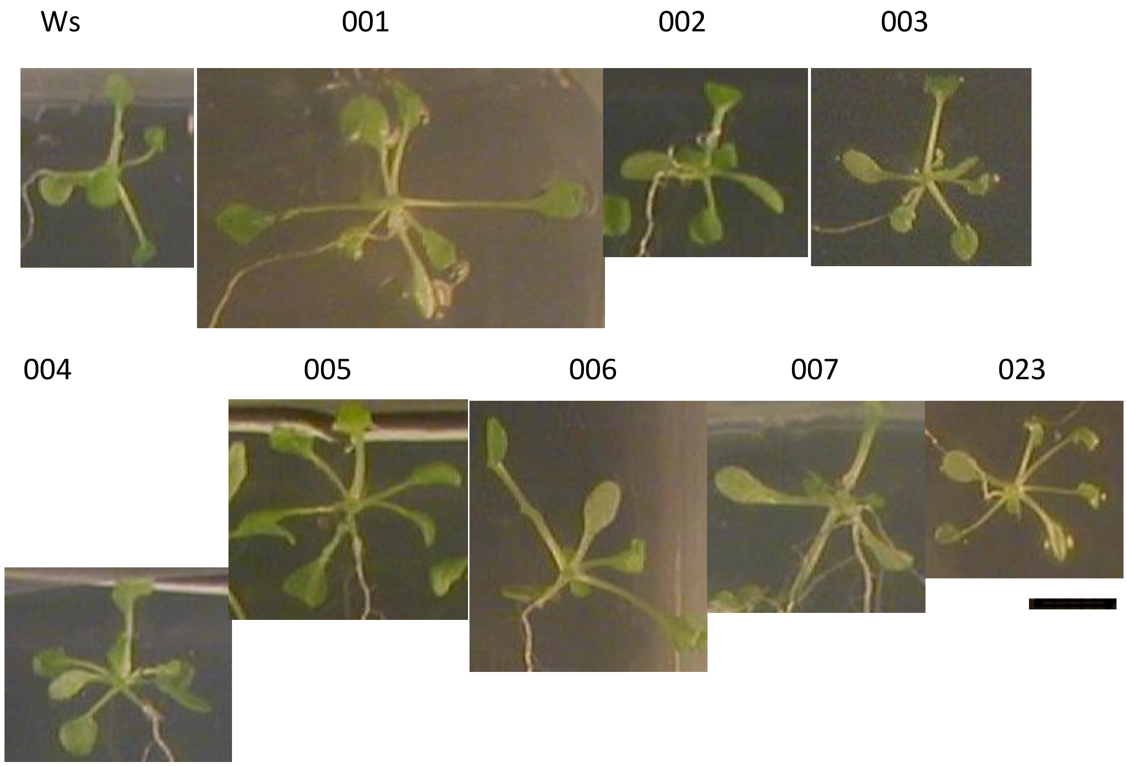
Ws
001
002
003
004
005
006
007
023
